# Community health workers for ART in sub-Saharan Africa: learning from experience – capitalizing on new opportunities

**DOI:** 10.1186/1478-4491-7-31

**Published:** 2009-04-09

**Authors:** Katharina Hermann, Wim Van Damme, George W Pariyo, Erik Schouten, Yibeltal Assefa, Anna Cirera, William Massavon

**Affiliations:** 1 Institute of Tropical Medicine, Department of Public Health, Antwerp, Belgium; 2 School of Public Health, Makerere University, Kampala, Uganda; 3 Department of HIV and AIDS, Ministry of Health, Lilongwe, Malawi; 4 Management Sciences for Health, Lilongwe, Malawi; 5 Federal HIV/AIDS Prevention and Control Office, Ministry of Health, Addis Ababa, Ethiopia; 6 Independent consultant, Barcelona, Spain; 7 Nsambya Hospital, Kampala, Uganda

## Abstract

Low-income countries with high HIV/AIDS burdens in sub-Saharan Africa must deal with severe shortages of qualified human resources for health. This situation has triggered the renewed interest in community health workers, as they may play an important role in scaling-up antiretroviral treatment for HIV/AIDS by taking over a number of tasks from the professional health workers. Currently, a wide variety of community health workers are active in many antiretroviral treatment delivery sites.

This article investigates whether present community health worker programmes for antiretroviral treatment are taking into account the lessons learnt from past experiences with community health worker programmes in primary health care and to what extent they are seizing the new antiretroviral treatment-specific opportunities.

Based on a desk review of multi-purpose community health worker programmes for primary health care and of recent experiences with antiretroviral treatment-related community health workers, we developed an analytic framework of 10 criteria: eight conditions for successful large-scale antiretroviral treatment-related community health worker programmes and two antiretroviral treatment-specific opportunities.

Our appraisal of six community health worker programmes, which we identified during field work in Ethiopia, Malawi and Uganda in 2007, shows that while some lessons from the past have been learnt, others are not being sufficiently considered and antiretroviral treatment-specific opportunities are not being sufficiently seized.

In particular, all programmes have learnt the lesson that without adequate remuneration, community health workers cannot be retained in the long term. Yet we contend that the apparently insufficient attention to issues such as quality supervision and continuous training will lead to decreasing quality of the programmes over time. The life experience of people living with HIV/AIDS is still a relatively neglected asset, even though it may give antiretroviral treatment-related community health worker programmes better chances of success than their predecessors and may be crucially important for adherence and retention in large-scale antiretroviral treatment programmes.

Community health workers as a community-based extension of health services are essential for antiretroviral treatment scale-up and comprehensive primary health care. The renewed attention to community health workers is thus very welcome, but the scale-up of community health worker programmes runs a high risk of neglecting the necessary quality criteria if it is not aligned with broader health systems strengthening. To achieve universal access to antiretroviral treatment, this is of paramount importance and should receive urgent attention.

## Introduction

Despite significant progress in scaling up antiretroviral treatment (ART) in low- and middle-income countries in recent years, the gap between the need for ART and the numbers currently receiving it is still wide in most of sub-Saharan Africa [1,2].

The health care systems of low-income countries with high HIV prevalence have been struggling to provide even basic health care to the population, let alone to deal with the additional burden of scaling up ART [3-5]. Moreover, ART poses a fundamentally new challenge for weak health systems, as it is transforming HIV/AIDS from a deadly disease into a chronic condition for which millions of people will need lifelong care. In the majority of low-income countries with high HIV prevalence in sub-Saharan Africa, the most crucial bottleneck for scaling up ART and managing an effective system of chronic ART care is the lack of qualified human resources for health (HRH) [4,6,7].

While estimations of HRH needs for scaling up ART show wide variations depending on contexts and programme variables [8], there is an enormous mismatch between the HRH needs of the prevalent ART delivery models and the HRH supplies in the health systems in most of sub-Saharan Africa [9-11]. This situation has triggered renewed interest in task shifting, as this approach may help to reduce the need for highly qualified health professionals in ART programmes [12,13]. According to WHO, task shifting describes the reallocation of certain tasks from more-specialized to less-specialized health care workers through the entire spectrum from the physician to the non-professional health care worker [13].

In this article we focus on task shifting for ART to community health workers (CHWs), asking how far they have taken on board the lessons learnt from past experiences with CHW programmes for primary health care and how far they are seizing the new HIV/AIDS-specific opportunities. Our framework for analysis is a list of 10 issues: eight conditions for successful large-scale CHW programmes plus two ART-specific opportunities.

We have opted for the term CHWs because it illustrates better than the terms lay providers or non-professional health care workers that the use of this type of cadre has a history that may provide important lessons for today. It is also widely used in the recent literature on task shifting and HRH issues in the scale-up of priority interventions such as ART [12,13]. We regard CHWs as lay people who have been trained in order to be able to assist the health professionals and to take over certain tasks from them. In doing this we acknowledge that we are not taking into account part of the original concept of CHWs, which emphasizes their role in community empowerment. This is one consequence of an important choice we made when conceiving the argument of the paper: We view CHW programmes exclusively from the perspective of the formal public health system, which results in some limitations regarding the complexity of CHW-related issues.

In the first part of our paper we establish the list of 10 criteria for successful CHW programmes for ART, which is based on our literature review of task-shifting, on previous multi-purpose CHW programmes for primary health care and on the more recent specific HIV and ART-related CHW programmes. In the second part we give six examples of ART-related CHW programmes, which we identified during our field research in Ethiopia, Tanzania and Uganda in 2007. Finally, we appraise the six CHW programmes according to our list of 10 criteria and formulate a conclusion.

## Task shifting to CHWs

Studies of the effectiveness of CHW programmes in sub-Saharan Africa in the past show a mixed picture. There is wide agreement on the potential of CHW programmes to improve access to and coverage of communities with basic health services. There is some evidence, too, that they can improve health outcomes under certain conditions [14-17]. But it has also been illustrated that many CHW programmes have not been successful. Especially large-scale and national CHW programmes have been beset by problems affecting their sustainability and the quality of services they provide [18,19].

Yet examples exist of large-scale programmes that are widely considered to be successful. One such example is the CHW programme set up by the Bangladesh Rural Advancement Committee (BRAC) in Bangladesh in the 1970s, which had expanded to more than 70 000 female CHWs by 2007. Taking a long-term view, BRAC has evolved the programme based on accumulating experience and learning [20]. Another programme that seems to be successful is the Brazilian *Programa Agente Comunitario de Saude *("Community Health Worker Programme"), with a coverage of more than 60 million people [21].

From our literature analysis it emerges that there are several fundamental characteristics of successful CHW programmes, just as there are some fundamental problem areas. Successful CHW programmes fulfil a number of conditions to ensure performance with regard to quality assurance, long-term reliability and scale-up of activities. We consider eight issues as essential for the success of CHW programmes: five of them are basic conditions for all CHW projects and three are necessary for the scale-up to large programmes with wide coverage. The success of a CHW programme depends on all eight conditions, and the neglect of even one may jeopardize the success of the entire CHW programme.

### 1. Selection and motivation

There is wide agreement that CHWs should be selected on the basis of their motivation to serve the community they will be working in. Belonging to this community is crucial. Prior level of education is less important, although literacy and numeracy facilitate participation in training and follow-up activities [16].

Selection that has not been carefully considered can lead to a lack of trust from the community and become a contributing factor to a high turnover of CHWs, which will make sustained quality assurance unlikely [16,22].

### 2. Initial training

This is of crucial importance and its length and content depend on the prior knowledge and the tasks and roles to be fulfilled by the future CHWs. Training should be practically oriented and not consist of transferring disease-specific knowledge alone, but also communication and counselling skills [14,22]. Guidelines and standardized protocols are beneficial tools for initial training.

### 3. Simple guidelines and standardized protocols

The use of protocols and standard guidelines is increasingly being recognized as an important tool for quality assurance in most health professions. CHWs are certainly no exception [14,16,23]. BRAC's CHWs, for example, who follow simple and standardized protocols for acute respiratory disease control, have received very positive evaluations [20]. Under this condition also fall issues related to the scope of practice and clear definition of the roles of CHWs.

Evaluations of PHC-CHW programmes in the past have shown that oftentimes CHWs were overwhelmed by a very broad range of tasks with negative effects on the overall quality of their performance. Also, CHWs with too many tasks tended to select only a few activities that they themselves regarded as most feasible. Clearly defined roles and standardized protocols should make sure that CHWs practise within the limits of what they can achieve and what they have been trained for. Simple guidelines and standards also greatly facilitate supervision and supply management.

### 4. Supervision, support and relationship with the formal health services

Especially supervision and other forms of support, such as supplies, are widely acknowledged in the literature as crucial for the continued quality of service provision by CHWs. Particularly large-scale CHW programmes have often neglected these areas, mainly because they had overlooked their cost in the planning stage [19,24-26]. Only good supervision, together with adequate material support, will enable CHWs to function. This can be organized through the formal public health system (e.g. the *Programa Agente Comunitario de Saude *in Brazil) or through a formal NGO network (e.g. BRAC in Bangladesh), but in both cases referrals to the formal health services need to be facilitated.

Also of crucial importance for sustaining the quality of performance of CHWs is continued support in terms of refresher training and regular mentoring. Several studies have shown that without refresher training, acquired skills are quickly lost [22,25].

Many instances of past CHW programmes have been described in which professional health care workers saw community members as lowly aides and failed to understand the potential value of their contribution. Thus the relationship between CHWs and the formal health services often became strained, negatively affecting the satisfaction and performance of CHWs [12,14,25]. To avoid this, the management of CHW programmes must also pay attention to the concerns and attitudes of health professionals [27].

### 5. Adequate remuneration/career structure

One major socioeconomic challenge that has been the subject of ongoing debate is the issue of payment versus voluntarism. The initial idea of the CHW assumed the existence of a pool of willing volunteers, but lack of payment has emerged as an important cause of attrition of CHWs in many programmes [16,26]. This is not to deny that much true voluntarism can be found in many communities, where people dedicate part of their time to social activities. Still, in truly voluntary programmes, CHWs are able to work a maximum of only a few hours per week and a high turnover of volunteers is the rule [27]. Most successful CHW programmes have therefore ensured that their CHWs receive adequate remuneration if their programme activities prevent them from gaining their livelihood in other ways [20,21]. Some evidence suggests that the possibility of professional development is an important motivating factor for CHWs, possibly improving retention [24,28].

Three additional conditions for the successful scale-up of CHW programmes are:

### 6. Political support and a regulatory framework

For national CHW programmes it is necessary to develop regulatory frameworks that demarcate the boundaries between CHWs and the professional health cadres and provide protection for patients as well as for health care workers [12]. Depending on the context, any of the above criteria can be part of the regulation: selection, training, supervision and support, and remuneration and career tracks.

### 7. Alignment with broader health system strengthening

As Abbatt points out, training large numbers of CHW will not be a "quick win", as implied by the United Nations Millennium Project report in 2005, as long as it is not accompanied by broader efforts to strengthen health systems [25]. Indeed, CHWs are not a remedy for weak health systems. Health systems must assure a number of functions, such as clinical care, uninterrupted supply, training and supervision, monitoring and evaluation, etc. CHWs can never be a substitute, but only an additional component in health systems that reliably fulfil these functions [27,29].

### 8. Flexibility and dynamism

There is some indication that in order to be sustainable and remain relevant, CHW programmes need to evolve in continuous interaction with the formal health system and, more widely, with the society they are based in. As patterns of societies are changing and health systems are becoming increasingly pluralistic, CHW programmes should not remain static but need to be reactive to newly arising needs, changing expectations and other evolving challenges [20].

## CHWs in the times of ART

It is becoming ever more obvious that for scaling up ART to the millions in need, not only the roles of professional health care workers must be redesigned but also the pool of other, non-professional HRH must be tapped [15,30]. Already, a wide variety of CHWs are active in many ART delivery sites. Thus, for example in our study of task-shifting practices in Ethiopia, Malawi and Uganda, we could identify at least six different types of CHWs in Ethiopia, six in Malawi and eight in Uganda.

In general, we can distinguish between CHWs who have long been established for a variety of health care activities and who have recently reassumed additional HIV/AIDS-related tasks, and those CHWs who have been especially introduced for specific HIV/AIDS-related tasks, such as serving as counsellors and expert patients. The majority of ART-related CHW programmes are not well documented and there is so far no systematic assessment of their performance and their potential to mitigate the HRH crisis. There are, however some studies that indicate that CHWs can make a positive contribution to the performance of ART programmes.

In Malawi, for example, Zachariah et al. describe the very positive experience of involving community volunteers in programme-related activities, such as, for example, voluntary counselling and testing (VCT), ART adherence counselling and referrals for ART or opportunistic infections [15]. The crucial role of CHWs for the success of the HIV/AIDS programme of Partners In Health in Haiti has been described at various stages of programme development, most recently by Mukherjee et al. in 2007 [31]. The CHWs (*accompagnateurs*) in Haiti are involved in many HIV/AIDS and TB-related activities, including even the provision of ART to the patients.

In Zambia, a study of the effectiveness of adherence support workers (ASWs) in adherence counselling, treatment retention and addressing HRH constraints at health facilities showed a marked shift of workload from health care workers to ASWs without any compromise in the quality of counselling. The loss to follow-up rates of new clients declined from 15% to 0% after the deployment of ASWs [32].

The AIDS Support Organisation (TASO) in Uganda has been working with lay providers, called "field officers", providing ART at home since June 2004. Adherence to ART has been shown to be very high and a recent study of the mortality under ART in this programme concluded that "the overall effect of ART on mortality was similar to or better than that seen in facility-based studies (...)" [33,34].

Based on such examples and on experiences with chronic care in high-income countries, we hold that in addition to the eight general conditions for successful CHW programmes, there are two more specific opportunities for ART-related CHW programmes, completing our list of ten issues:

### 9. Using the life experience of People Living with HIV/AIDS (PLHAs)

What makes HIV/AIDS special is that it is a chronic condition resulting in a growing pool of people living with the disease. The concept of using the personal experiences of people living with the disease is emerging as one important building block for chronic care programmes in industrialized countries [35-38]. The National Health Service of the United Kingdom went furthest in establishing an expert patients programme as one pillar of the national chronic disease management programme [36,39]. Here, people living with the disease are involved as volunteers in training and counselling activities and their life experience is regarded as their most important asset [40]. We judge the potential of using the life experience of PLHAs in ART models as very promising and thus worth further exploration in CHW programmes.

### 10. Using chronic-care models, with their special focus on adherence to treatment and retention in care

Chronic-care models usually put a lot of emphasis on the self-management skills of patients in order to achieve the best results in terms of adherence to treatment and long-term retention in care. The problems of loss to follow-up and the negative effects of non-adherence are well documented for ART programmes [41,42]. We regard adherence and retention in care as two of the most important issues for the long-term success of ART programmes and contend that PLHAs are probably best qualified by their life skills to promote these.

## Examples of ART-related community health workers in Ethiopia, Malawi and Uganda

Of the three countries, it is Uganda where we met with the widest range of CHWs involved in ART-related health activities. We identified the following eight types, which may not be exhaustive: ART aides, HIV medics, field officers, community ART and TB treatment supporters (CATTS), community AIDS support agents (CASAs), AIDS community workers (ACW), expert patients (network support agents) and TB tracers. According to our knowledge, only the field officers have been described in various publications [33,43,44]. We present here four types of CHWs whom we found to be most involved in ART-related services: expert patients, ART aides, HIV medics and field officers. None of these four types of CHWs is formally recognized or regulated by the Ministry of Health (MoH).

Expert patients are found in almost every ART site in Uganda. They are by no means a clearly defined group or cadre, as the characteristics of their recruitment, their training, their responsibilities and their remuneration depend on the respective NGO that is locally in charge of the expert patient programme. Accordingly, their salary ranges from less than USD 2 to USD 75 per month. The main common selection criterion is their positive HIV status. The most generally known "expert patients" are TASO's Network Support Agents, who receive five weeks' training in VCT and two weeks' training in ART-related tasks. While the term "expert patients" is clearly being used as a label for these and other HIV-positive lay providers, we did not find that the term had the same meaning as the original concept of the expert patient, as it was developed for the self-management of chronic disease care [36].

ART aides are mostly but not necessarily PLHAs, trained in five days with the WHO Integrated Management of Adolescent and Adult Illness (IMAI) course by the NGO Uganda Cares. Most of the more than 20 ART aides in 2007 were chosen from among PLHAs who had received previous training as expert patients, also as part of the IMAI approach. The training of ART aides is focused on general support for HIV care and ART, with specific activities in triage, adherence support, group education, pre- and post-test counselling, drug dispensing and records management at health centre level. The ART aides receive a salary of USD 35 per month from Uganda Cares.

HIV medics are trained by the NGO Uganda Cares and supported by the AIDS Healthcare Foundation. They are a mix of PLHAs on ART (about 25%) and non-PLHAs with no prior medical background. They are required to have a high school education and be able to read and write English. They follow a 12-week training course of which the curriculum includes six weeks of classroom teaching and six weeks of practical clinical training. It covers topics such as general knowledge of HIV/AIDS and ART, counselling, adherence support, medical history-taking, triage, examination and referral of patients and follow-up of patients. Some HIV medics have additionally been trained in doing CD4 tests and HIV tests. By June 2007, 55 participants had completed the course and were employed and paid by different NGOs. The ones employed by Uganda Cares receive a salary of USD 226 per month.

Field Officers are mostly social workers with a university degree. They are employed by TASO. Their training lasts around two months and enables them to follow up clients on ART at home, including the delivery of ARVs, provision of home-based care and counselling and referral of complicated patients. They are selected by TASO centres and supervised monthly by the Parish AIDS Committee. They receive a monthly salary of about USD 350 and a daily lunch allowance of about USD 3. With their high level of education, they are fairly atypical CHWs.

In Malawi we identified the following six types of CHWs involved in HIV-related activities: community health workers, community care providers, VCT community counsellors, volunteers trained at the health facilities, HBC volunteers and health surveillance assistants (HSAs). We chose to focus on the HSAs, as they are the most widely established. Also, while there is some literature on HSAs [15,24], we found none on any of the other CHWs.

HSAs have been in existence since the 1960s and 1970s, when they were recruited as temporary "smallpox vaccinators" and "cholera assistants". Malawi's Ministry of Health and Population (MoHP) decided to keep these trained people for the purpose of surveying health risks and providing basic care before referral to a health facility. Over the years the mandate of HSAs has widened considerably and now includes vaccination of under-fives, growth monitoring, supervision of traditional birth attendants, sanitation, water source protection and water treatment, disease surveillance, health and nutrition advice, provision of family planning devices and the follow-up of TB patients [24]. While they were a cornerstone of the preventive health care system, it was not until 1995 that HSAs became officially regulated as part of the structure of the MoHP, from which they also receive a salary, ranging between USD 42 and USD 52 per month.

In the context of the HIV/AIDS programme and the scaling-up of ART in a number of projects and districts, the HSAs have been assigned a number of additional tasks, such as HIV prevention, provision of VCT, basic care for opportunistic infections, administration of cotrimoxazole prophylaxis, ART defaulter tracing, prevention of mother-to-child transmission for the newborn and general support to ART clients. However, we found that the specific tasks given to HSAs differed greatly in the various facilities studied. Their HIV/AIDS-related roles and functions were determined by the level of resources available and the needs at each site.

The initial training for HSAs lasts 10 weeks and focuses on their core tasks. Training for HIV-related activities is shorter and occurs after the initial training. HSAs are selected centrally. After training they are sent to the communities in which they are to work and live.

While in 2004 there were around 4000 HSAs in the country, by 2008 their number had almost tripled, to nearly 11 000. This fast expansion was made possible with funding from the Global Fund to Fight AIDS, Tuberculosis and Malaria, but the formal training of new staff has not yet taken place and is being replaced by on-the-job initiation by NGOs or existing HSAs.

In Ethiopia we identified six different types of CHWs involved in HIV-related activities: health extension workers (HEWs), care givers/care aides, expert patients, Kebele health workers, community counsellors and community health agents [45].

We want to focus here on the HEWs, because the Government of Ethiopia is investing substantially in a Health Extension Programme for increasing the access of the population to promotive, preventive and curative care. Also, there are number of publications focusing on HEWs [28,46,47], but we did not find anything specifically on the other types of CHWs.

The cadre of HEWs was created in 2003; by the end of 2007 more than 17 600 people had been trained. There are now 24 000 HEWs, and the aim is to increase their number to 30 000 by 2009 [48]. HEWs must be female and must have a high school education. They must be members of the community they will serve in and they are selected by a committee of the local administration (different Woreda offices).

Their training lasts one year and includes theoretical as well as practical background, covering a wide array of mainly promotive and preventive topics within the four categories of hygiene and environmental sanitation, family health services, disease prevention and control and health education and communication.

According to their job description they spend 25% of their time in the health posts and the other 75% in the community. HIV/AIDS is part of the curriculum, and we have identified the following activities of HEWs: provision of HIV education; psychological support; HIV counselling; prevention of mother-to-child transmission of HIV, including the provision of Nevirapine; patient care during home visits; ART adherence counselling; individual or group treatment support; referrals of complicated patients; and defaulter tracing [49]. HEWs are part of the national Ethiopian health service, receiving a monthly MoH salary equivalent to USD 68.

## Appraisal of ART-related CHW programmes

Based on what we know about the CHW programmes described in the previous section, we want to attempt to examine them against the background of the eight conditions for the success of past CHW programmes and the two HIV/AIDS-specific opportunities.

### 1. Selection and motivation

The ART aides and HIV medics in Uganda are selected and recruited by NGOs or the health facilities. Also, the field officers are selected by TASO and not by the community, but it should be noted that they are to some extent part of a wider community-based structure. Thus, TASO's AIDS community workers and community AIDS support agents are usually identified in dialogue between the programme managers and the communities. It is the communities themselves that decide on their final selection. The HSAs in Malawi must live in the community and profess the motivation to serve the community they will be working in. Their selection, however, is done centrally and not by the community. Only the HEWs in Ethiopia are selected with the participation of the community. Our finding that communities are not necessarily pivotal in the selection of CHWs may be related to the fact that some of the cadres reviewed are rather facility-based lower cadres than real community-based health care workers. Of all six programmes, only the expert patients and ART aides are chosen on the basis of having a positive HIV status.

### 2. Initial training

It is a matter of course for all six CHW programmes to provide initial training to the prospective CHWs. The length and type of initial training vary between programmes and it is not the purpose of our overview to assess its quality or adequacy. However, the example of the HSAs in Malawi indicates that the timely provision of adequate training can become a challenge. Recently, this cadre was vastly expanded, from 4000 to 11 000, but the plans for initial training in HIV-related tasks have not yet been realized. The new HSAs are still being trained on the job by the existing HSAs and by local NGOs. The 12-month-long training of HEWs in Ethiopia may well prove one important factor of success.

### 3. Simple guidelines and standardized protocols

In the four Ugandan programmes created exclusively for HIV/AIDS-related care, the CHWs adhere to a relatively narrow range of activities. HIV medics and ART aides, for example, are given very specific tasks at the laboratory, the pharmacy and the consultation room of the health facilities. By contrast, the HSAs and HEWs, who are working in much broader community health programmes, must fulfil a much larger range of tasks. Interviews with HSAs in Malawi revealed that many of them feel overloaded with work, as more and more tasks are being added to their job description. This was also seen as one of the reasons affecting the quality of their performance in key activity areas such as immunization [24]. Judging from past experiences with PHC-CHWs, this very broad range of tasks may overstrain the CHWs in the national programmes in Malawi and Ethiopia.

### 4. Supervision, support and relationship with formal health services

Responsibility for the supervision of ART aides, HIV medics and expert patients in Uganda lies with the respective health facility where the CHWs are based. The ART aides should be supervised by HIV medics, the home-based care coordinator or a health centre nurse; the HIV medics should work under the supervision of physicians, nurses or clinical officers [50]. These CHWs conduct their main activities at the health facilities, and close and daily contact with the professional health care workers facilitates supervision. The supervision rules for expert patients depend on the health facility or the NGO where they are employed.

The HSA programme in Malawi prescribes that HSAs should be supervised by environmental health officers and community health nurses. The survey by Kelly et al. described the actual supervision system as inadequate and reported that due to transport problems, supervision hardly ever occurred except on immunization days, when transport was available [24]. In the same survey, the HSAs also complained about lack of transport and irregular supply of drugs and vaccines. In view of the decreasing HRH base and increasing workload due to HIV/AIDS in Malawi, the issue of insufficient supervision and support looks likely to remain very problematic in the years to come.

The HEWs in Ethiopia are in most cases supervised by the Woreda Health Office and sometimes also by the health centre where they are based. An assessment by the Center for National Health Development in Ethiopia from May 2006 found that good guidelines for team supervision exist and that a lot of attention was given to the supervision of HEWs at all levels. However, the Woreda Health Offices as well as the health centres were usually neither sufficiently staffed nor trained to provide good supervision [28].

It seems that in none of the programmes has the issue of refresher training received much attention in the initial planning process. Uganda, for example, had a well-organized network of community-based health care NGOs in the past, who variously developed criteria and trainer and facilitator manuals. But these have not been taken up by the new ART-oriented CHW programmes, except in those supported by TASO. Given the importance of continuing training for a sustained quality of service provision by CHWs, there is a risk that this may become a weakness of these CHW programmes.

While in small CHW projects with strong NGO back-up the organization of sufficient support looks feasible, it is much more of a challenge for the large national programmes. There are major doubts about adequate supervision and support in these programmes, especially due to the overall lack of professional HRH. Also, clinicians are usually poorly trained for such tasks and the relationship between health professionals and CHWs may become strained because of frustrated expectations on both sides. There is a real risk that poor supervision and support will compromise the quality of the large-scale CHW programmes.

### 5. Remuneration and career structure

In all six programmes the CHWs receive a regular salary. TASO's field officers earn a monthly USD 350 and the HIV medics earn – at USD 226 per month – only a little less than even a nursing or a clinical officer. Although the pay of ART aides is quite modest, at USD 35 a month, given the wide-scale rural unemployment it may constitute an important reason to continue service as a CHW. It is quite striking that there is such a wide range of salary options for CHWs with activity packages that do not differ greatly. In Malawi and Ethiopia, where the HSAs and HEWs are part of the MoH structure, their salary is below that of the professional health care workers.

None of the CHW cadres in Uganda has so far been formally recognized by the MoH. The consequence is that they do not have structured career opportunities. A recent policy prescribes that there should be village health teams with the role, among other things, of selecting and supporting CHWs. The modalities of how this will actually operate are still under development, leaving room for various NGOs to experiment with different forms of CHW programmes.

The HSAs in Malawi, by contrast, have a career path. According to the Ministry, they can be promoted to the position of senior HSA; plans have been made recently to create several levels of HSAs with increased salary scales. They also have a better chance of being accepted for further studies to become environmental health officers, clinical officers or nurses.

The HEWs in Ethiopia have an opportunity to upgrade to nurses. This depends on their performance and recommendation from their supervisors. However, by 2008 none of the HEWs had so far upgraded.

### 6. Political support and regulatory framework

As the CHWs in Uganda are not officially recognized by the MoH they do not have a regulatory framework, despite working in MoH facilities. A system-wide scale-up of one specific CHW programme for the provision of ART does not seem to be intended. The HSAs in Malawi and the HEWs in Ethiopia are officially regulated by the Ministries of Health. In fact, in both countries it was the MoH, supported by donors, that decided to quickly and substantially expand these cadres.

### 7. Alignment with broader health system strengthening

This point can be regarded as a summary of most of the previous points. The national scale-up of a CHW programme for ART is conceivable only in a strong health system that can provide regular follow-up training, organize and sustain adequate support and supervision, ensure adherence to protocols and implement and enforce a regulatory framework. CHWs are not a substitute for professional HRH, but only a complement.

### 8. Flexibility and dynamism

All programmes are reactions to the new challenges posed by HIV/AIDS and the scale-up of ART. The Ugandan CHWs have been newly created for HIV-related purposes; the Malawian and Ethiopian CHWs have been assigned new HIV-related functions. How far these CHW programmes will interact in flexible and dynamic ways with the formal health services and evolve along with broader changes in the societies, remains to be seen.

### 9. Using the life experience of PLHAs

We have mentioned that only two small-scale NGO projects select their CHWs on the basis of being PLHAs. Of course, there exist many other smaller projects in all three countries, such as mutual support groups, peer educators and community counsellors that specifically involve PLHAs, with good results. However, neither of the two large-scale national programmes uses this "positive discrimination" of PLHAs in their selection of CHWs. Not to tap the life experience of the ever-growing pool of PLHAs on ART means missing an important new HIV-related opportunity.

### 10. Chronic-care focus on adherence and retention

Small-scale NGO projects, such as those described in Uganda, often pay high attention to the issues of adherence and retention in care. We have the impression, though, that these two crucially important aspects of long-term success of ART programmes have so far been relatively neglected in the large-scale national CHW programme in Malawi. Ethiopia has recently pilot-tested a case management programme as a strategy to provide a continuum of care and link the health facilities with the community to prevent loss to follow-up and improve adherence to treatment. The plan is to scale up the case management programme at national level once it is evaluated [51]. However, the involvement of PLHAs in tasks such as adherence counselling and defaulter training has not been considered, even though it may be one of the most important elements for achieving good results in these two crucial programme aspects.

## Conclusion

Our appraisal of the CHW programmes in Uganda, Malawi and Ethiopia shows that some lessons seem to have been learnt from past experiences but that others have been neglected and that important weaknesses remain. New ART-related opportunities are not sufficiently seized.

All programmes have learnt the lesson that CHWs cannot be retained in the long term if they do not receive adequate remuneration. Yet concerns about the long-term funding of NGO programmes with high CHW salaries have been voiced.

Based on lessons from the past, we contend that while an adequate and competitive salary may prevent a high turnover of CHWs, the apparently insufficient attention to other issues such as quality supervision and continuous training will lead to decreasing quality of the programmes over time. The strong need for support and training illustrates clearly that CHWs are not a simple and cheap solution to the lack of qualified HRH. CHW programmes that seem to be successful show that quite the contrary may be the case: they usually employ many qualified HRH for training, supervision and support [15,52]. Therefore, the real cost of scaling up CHW programmes, including the additional qualified HRH for supervision and training, should not be neglected.

The government programmes seem more attractive than the NGO-based programmes for scaling up ART and reaching coverage, as the CHWs are already part of the health system's structure, regulatory frameworks exist and career prospects can be created. However, we contend that they run the highest risk of neglecting quality assurance if their scale-up is not aligned with broader health systems strengthening. For scaling up ART, health systems need to fulfil many functions in a reliable way, including the provision of support, supervision and training of CHWs. Therefore, CHWs can only ever be an addition, never a substitute for health systems strengthening [27,29].

We have the impression that small NGO projects are more likely than large national programmes to select PLHAs as CHWs. Not to capitalize on the life skills of the growing number of PLHAs for the crucial programme aspect of long-term retention in care is a missed opportunity of large-scale CHW programmes. It is easy to imagine how much more motivating it would be, for example, for an HIV-positive pregnant woman to be counselled by an HIV-positive mother with a healthy child than by a CHW without this personal experience and with only limited training.

We argue that current CHW programmes for ART should not be regarded as something entirely new but as standing in the context of a history of CHW programmes, so that lessons of failure and success, as outlined here in the form of eight conditions, can be incorporated in the design of new CHW programmes. The use of the life experience of PLHAs may give HIV/ART-related CHW programmes better chances of success than their predecessors and may be crucially important for adherence and retention in large-scale ART programmes [35].

Due to our formal health system perspective, we did not deal with an important aspect of the original CHW concept, i.e. their role as agents of change in the relationship between health services and population and for community empowerment. More research on non-facility-based CHW programmes, their lessons of failure and success, and their present and potential role in the scale-up of ART, would be very useful and timely.

CHWs as a community-based extension of health services are essential for ART scale-up and comprehensive PHC. The renewed attention to CHWs is thus very welcome, but the scale-up of CHW programmes runs a high risk of neglecting the necessary quality criteria. To achieve universal access to ART, this is of paramount importance and should receive urgent attention.

## Competing interests

The authors declare that they have no competing interests.

## Authors' contributions

KH reviewed the literature and drafted the manuscript. WVD conceptualized the study and reviewed the various drafts of the text. AC, WM and WVD designed and conducted the field studies. GWP, YA and EJS contributed country-specific data and reviewed the manuscript before submission. All authors have seen and approved the final version.
